# *FLCN* Multi‐Exon Deletion in a Chihuahua With Renal Cysts and Nodular Dermatofibrosis

**DOI:** 10.1002/age.70215

**Published:** 2026-09-29

**Authors:** Melvin Schneiter, Julie Selz, Jacky Jonker, Martina Dettwiler, Vidhya Jagannathan, Tosso Leeb, Elisa Maina

**Affiliations:** ^1^ Institute of Genetics, Vetsuisse Faculty University of Bern Bern Switzerland; ^2^ Medi‐Vet SA Lausanne Switzerland; ^3^ Vetscope Pathologie Dettwiler Riehen Switzerland; ^4^ Division of Clinical Dermatology, Vetsuisse Faculty University of Bern Bern Switzerland

Birt‐Hogg‐Dubé syndrome in humans is an autosomal dominant inherited disorder characterized by the development of hair follicle hamartomas, kidney tumours, and spontaneous pneumothorax. It is caused by heterozygous loss‐of‐function variants in the tumour suppressor gene *FLCN* encoding folliculin. A homologous tumour syndrome termed renal cystadenocarcinoma and nodular dermatofibrosis (RCND) was characterized in German Shepherd dogs more than 40 years ago (Moe and Lium 1984; Lium and Moe 1985) and is caused by a missense variant in *FLCN*, XM_005619508.3:c.764A>G or XP_005619565.1:p.H255R (OMIA: 001335–9615) (Lingaas et al. 2003). RCND caused by the same mutant *FLCN* allele has also been reported in a mixed‐breed dog (Ciccarelli et al. 2019).

An 11‐year‐old female Chihuahua was presented for multiple gradually increasing masses across the entire body since the age of 7 years, with faster growth observed in the oral masses (Figure 1). The owners reported that its sire had also developed nodules at an older age, but the sire was not available for investigation. Defecation was normal, but the oral masses occasionally interfered with eating. The dog developed left hindlimb lameness, progressing to non–weight‐bearing. In a general clinical examination, the dog had a body condition Score of 4/9, with normal hydration and Grade 4/4 medial patellar luxation. The dog showed severe atrophy of the left hind limb, multiple cutaneous and subcutaneous masses throughout the body which were hard in consistency, deeply adherent, hyperpigmented and varying in size from a few millimeters to several centimeters, and presence of a dense fibrous mass on the left side of the mouth. Radiographs showed cystic masses primarily on the posterior of the body and a dislocation of the patella. The histological examination of the oral lesion and the cutaneous and subcutaneous nodules revealed the masses to consist of well‐differentiated dense fibrous connective tissue, with mildly thickened collagen fibers and activated fibroblasts (Figure S1). Skin adnexal structures were displaced or atrophic, and occasional follicular cysts were present within the fibrous tissue. The oral mass was partially ulcerated. An abdominal ultrasound was performed, showing the kidney bilaterally enlarged with multiple bilateral cortical cysts, with the conclusion of a diagnosis of minimally progressive moderate to severe bilateral chronic polycystic nephropathy. Given the similarity of clinical signs with RCND in German Shepherd dogs (Lium and Moe 1985) an underlying genetic variant in the *FLCN* gene was suspected.

**FIGURE 1:**
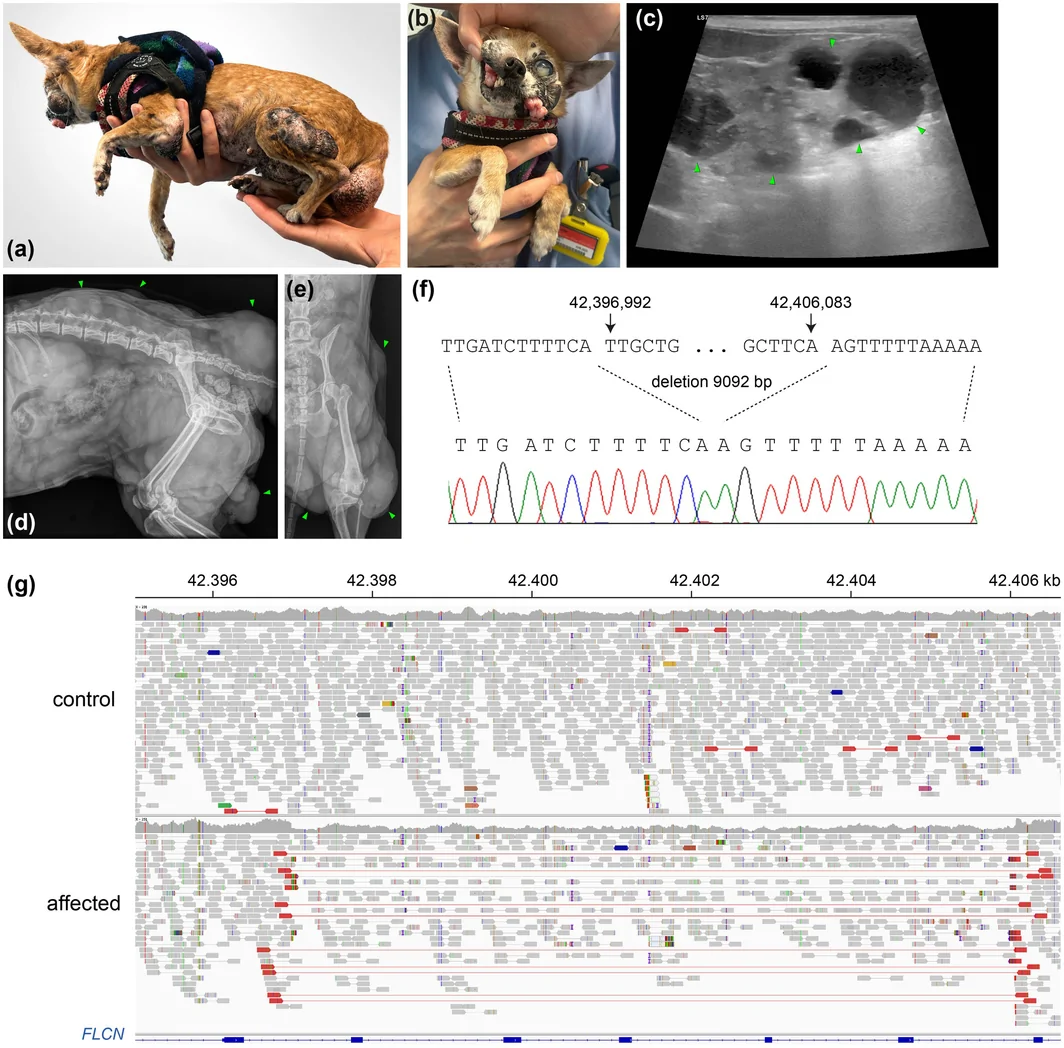
Clinical phenotype and genetic findings. (a) Whole‐body lateral view showing widespread, multifocal to coalescing alopecic and hyperpigmented cutaneous/subcutaneous nodules and plaques, with particularly large masses affecting the left facial/cheek region, medial left elbow, left thigh/femoral region and perineal/perianal area. Marked atrophy of the left pelvic limb is also evident. (b) Close‐up view of the head showing multiple oral and labial fibrous masses, as well as multifocal, firm, hyperpigmented cutaneous/subcutaneous nodules and plaques affecting the muzzle, lips, nasal planum and periocular skin. (c) Longitudinal view of the left kidney. Both kidneys showed multiple well‐defined round to ovoid cystic structures throughout the renal cortex and corticomedullary region causing distortion of the normal renal architecture and renal contour. The cysts contain anechoic to slightly echoic contents and are thin walled, ranging in size from 0.5 to 3 cm in diameter. No dilation of the renal pelvis is observed. (d) Lateral abdominal and zoomed (e) ventrodorsal pelvis and left hindlimb. The radiographs demonstrate multiple cutaneous nodular soft tissue opacities indicated by green arrowheads surrounding the lumbar region and the left hindlimb above the stifle joint (sigmoid‐shaped angular limb deformity involving the stifle joint unrelated to the described lesions). (f) Sanger sequencing of an amplicon spanning the deletion breakpoint defined the exact breakpoint of the genomic deletion. (g) IGV screenshot showing read‐alignments of a control dog (top) and the affected dog (bottom). The heterozygous deletion removing 5 of 11 coding exons of the *FLCN* gene is clearly indicated by a corresponding drop in coverage and read‐pairs that flank the deletion on both sides.

We isolated genomic DNA from an EDTA blood sample. The genome of the affected dog was sequenced with 2 × 150 bp chemistry at 17.5× coverage on an Illumina NovaSeq 6000 instrument. The generated reads were processed as described before (Jagannathan et al. 2019) and mapped to the UU_Cfam_GSD_1.0 reference assembly. The initial analysis confirmed that the affected dog did not carry the mutant G‐allele at the previously published *FLCN*:XM_005619508.3:c.764A>G variant. We then visually inspected the short‐read alignments of the affected dog and detected a heterozygous deletion of 9092 bp, harboring five coding exons of *FLCN*, NC_049226.1:g.42396992_42,406083del. This deletion removed 813 nucleotides or 47% from the coding sequence, NC_049226.1(NM_001127611.1):c.250‐750_1062 + 1289del. We confirmed the exact sequence of the mutant allele by Sanger sequencing of a PCR amplicon spanning the deletion breakpoints (Figure 1, Table S1). This deletion was absent from a sample of 1000 genetically diverse dogs with publicly available whole genome sequence data (Table S2).

Our study reports a case of RCND with striking clinical findings in a Chihuahua that was presumably inherited from its sire. Clinically, the concurrent occurrence of numerous firm cutaneous and subcutaneous fibrous nodules together with bilateral renal cystic disease strongly supported a diagnosis within the RCND spectrum. The oral fibrous mass may represent part of the same fibroproliferative process, although a reactive lesion secondary to chronic gingival irritation cannot be fully excluded. No overt renal carcinoma was detected by ultrasound at the time of examination; however, the renal cystic changes were considered clinically relevant in the context of the typical skin phenotype. Whole genome analysis identified a heterozygous multi‐exon deletion in the functional candidate gene *FLCN*, which represents a very plausible candidate causal variant for the observed phenotype. Haploinsufficiency of *FLCN* has been described to cause RCND in German Shepherd dogs and Birt‐Hogg‐Dubé syndrome in humans (Nickerson et al. 2002; Lingaas et al. 2003). Classification of the deletion according to the human ACMG/AMP or AVCG v2 criteria (Richards et al. 2015; Desmet et al. 2026) results in a classification of pathogenic (Table S3). To the best of our knowledge, the identified deletion represents the second *FLCN* variant in dogs that is associated with RCND. In this context, it is interesting to note that the literature reports several dogs with RCND (Gómez‐Barris et al. 2024; Lee et al. 2025) or isolated nodular dermatofibrosis (Zanatta et al. 2013), which were wildtype at the previously published *FLCN*:p.H255R variant. We speculate that other spontaneous *FLCN* variants may have caused the phenotype in these dogs. One study reported a Bichon Frise with typical clinical and histopathological findings of RCND (Lee et al. 2025). Whole exome sequencing in this dog confirmed the absence of the p.H255R variant and did not reveal any other *FLCN* variant (Lee et al. 2025). This led the authors to speculate about the potential contribution of *BRCA2* variants to the observed phenotype (Lee et al. 2025). We think that an alternative explanation might have been the existence of a heterozygous large structural variant in *FLCN*, such as in the Chihuahua described herein, which might easily have been missed during whole exome sequencing.

In conclusion, we characterized an advanced stage of RCND in an 11‐year‐old Chihuahua. The family history was consistent with autosomal dominant inheritance. The genetic analysis identified a heterozygous 9 kb multi‐exon deletion in the *FLCN* gene as the most likely candidate causal variant. To the best of our knowledge, this is the second pathogenic variant in *FLCN*, which has been characterized at the molecular level. Knowledge of this variant confirms allelic heterogeneity in RCND and should be taken into account during genetic testing.

## Author Contributions

**Melvin Schneiter:** investigation, writing – original draft, writing – review and editing. **Julie Selz:** investigation, writing – review and editing. **Jacky Jonker:** investigation, visualization, writing – review and editing. **Martina Dettwiler:** investigation, visualization, writing – review and editing. **Vidhya Jagannathan:** data curation, writing – review and editing. **Tosso Leeb:** conceptualization, supervision, visualization, writing – original draft, writing – review and editing. **Elisa Maina:** conceptualization, investigation, visualization, writing – original draft, writing – review and editing.

## Funding

The authors have nothing to report.

## Ethics Statement

The affected dog in this study was privately owned and blood samples for diagnostic purposes were collected with the consent of its owner.

## Conflicts of Interest

The authors declare no conflicts of interest.

## Supporting information


**Figure S1:** Histopathological findings.

**Table S1:** PCR primer details for amplifying the deletion breakpoint.

**Table S2:** Accession numbers of 1001 dog genome sequences.

**Table S3:** Variant classification.

## Data Availability

The genome sequence data of the affected dog was submitted to the European Nucleotide Archive (ENA) with study accession PRJEB16012 and sample accession SAMEA120918502.
