# Supplementary material for: FLCN Multi‐Exon Deletion in a Chihuahua With Renal Cysts and Nodular Dermatofibrosis
Source: Anim Genet. 2026 Sep 29;57(5):e70215. doi: 10.1002/age.70215 (PMC13621923; doi:10.1002/age.70215)
Supplement: Supplementary file 1 [file AGE-57-0-s003.pdf]

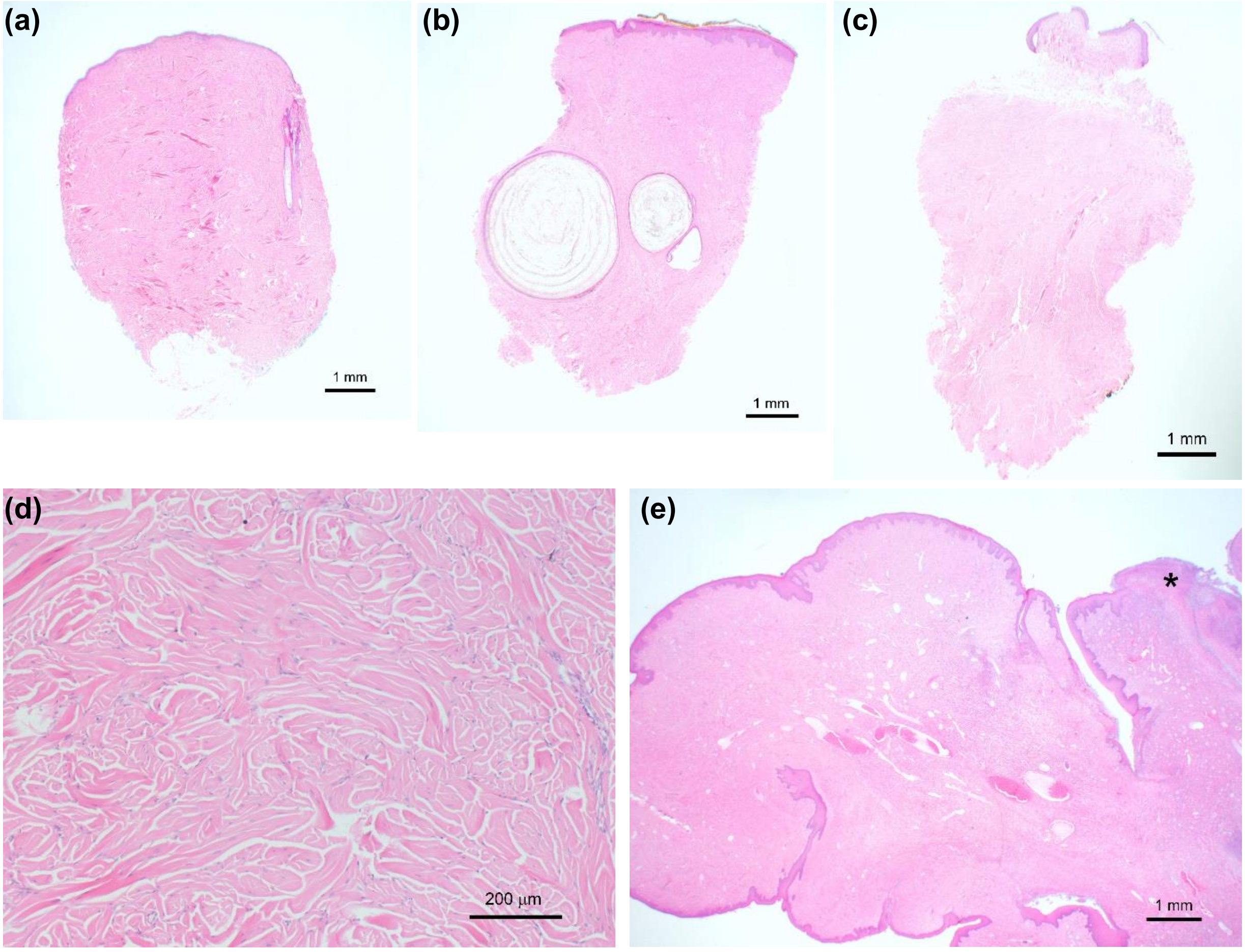

Figure S1. Histopathological findings (hematoxylin & eosin stain). Punch biopsies from the cutaneous nodules revealed multiple dermal (**a, b**) and subcutaneous (**c**) nodules consisting of dense fibrous tissue. Hair follicles are displaced (**a**) or incorporated within the nodules as follicular cysts (**b**). (**d**) The cutaneous masses are composed of broad interlacing collagen fibers admixed with activated fibroblasts. (**e**) The gingival mass is a lobulated, vascularized fibrous tissue mass covered with a hyperplastic epithelium showing focal ulceration (asterisk).
